# Synchrotron radiation circular dichroism spectroscopy reveals structural divergences in HDL-bound apoA-I variants

**DOI:** 10.1038/s41598-017-13878-z

**Published:** 2017-10-19

**Authors:** Rita Del Giudice, Oktawia Nilsson, Joan Domingo-Espín, Jens O. Lagerstedt

**Affiliations:** Department of Experimental Medical Science, Lund University, 221 84 Lund, Sweden

## Abstract

Apolipoprotein A-I (apoA-I) in high-density lipoprotein (HDL) provides cardiovascular protection. Synchrotron radiation circular dichroism (SRCD) spectroscopy was used to analyze the dynamic solution structure of the apoA-I protein in the apo- and HDL-states and the protein structure conversion in HDL formation. Wild-type apoA-I protein was compared to human variants that either are protective (R173C, Milano) or lead to increased risk for ischaemic heart disease (A164S). Comparable secondary structure distributions in the HDL particles, including significant levels of beta strand/turn, were observed. ApoA-I Milano in HDL displayed larger size heterogeneity, increased protein flexibility, and an altered lipid-binding profile, whereas the apoA-I A164S in HDL showed decrease thermal stability, potentially linking the intrinsic HDL propensities of the variants to disease risk.

## Introduction

Apolipoprotein A-I (apoA-I) is the main protein component of high-density lipoprotein (HDL) and carries cholesterol and other lipids in blood circulation. The apoA-I protein has a central role in the so-called reverse cholesterol transport pathway where excess cholesterol is removed, e.g., from the vascular wall, and transported back to the liver. This has been described as the major atheroprotective effect of apoA-I^1^. Other beneficial effects of the apoA-I protein, including its anti-thrombotic and anti-oxidant functions, and a positive influence of apoA-I/HDL on glucose control, with potential implications in the prevention and treatment of diabetes, have also been demonstrated^2–6^. These features of the apoA-I protein have spurred large interests in exploring the translational medicine potential in cardiovascular disease and diabetes^7,8^.

Much attention has also been given to the structural and functional properties of the apoA-I protein, both in the lipid-free and lipid-bound states. Despite apoA-I is a relatively small protein (243 amino acids in the mature protein), it is highly abundant in blood (about 140–160 mg/dl apoA-I^9^) and can be easily produced in large amounts as recombinant protein from heterologous production systems, its high structure plasticity and large number of hydrophobic amino acids have challenged classical X-ray crystallography approaches. However, X-ray structures from terminally truncated apoA-I proteins have been successfully obtained^10,11^. Additional important advances in the structure/function knowledge of apoA-I have been achieved by complementary approaches such as cross-linking combined with mass-spectroscopy^12–16^, hydrogen-deuterium exchange^17^, and electron paramagnetic resonance (EPR) spectroscopy^18–20^. For example, the bottom-up EPR approach where information on the local milieu (chemical and sterical) of individual amino acid side chains are obtained has provided secondary structure maps of lipid-free apoA-I and discoidal 9.6 nm diameter HDL^18,20^ and cross-linking experiments have defined intra- and inter-peptide distances in the apoA-I protein^12–16^. Still much remains to be defined including the apoA-I protein structure dynamics in the lipid-binding process and how the secondary structure elements (alpha helix, beta strand/turn and random coil) of apoA-I changes, as well as determination of high-resolution structures of the different states of the apoA-I protein.

In addition to native apoA-I, several human variants of apoA-I that affect the *in vivo* function of the protein have been described. Among them, the most well-known is the protective Milano variant where the arginine at position 173 is substituted for a cysteine (R173C). It was shown that individuals carrying this variant have normal, or better, protection against cardiovascular disease despite low levels of HDL^21,22^. Formulations based on the Milano apoA-I protein is tested in clinical trials for coronary artery disease^23^. On the contrary, an apoA-I variant that was recently identified in a Danish cohort (10,330 individuals of the general population), the A164S variant (alanine at position 164 is substituted by a serine), was shown to increase the risk of ischeamic heart disease and mortality despite normal levels of apoA-I and HDL^24^. The molecular basis for how these seemingly minor modifications can have such significant impact on *in vivo* function and disease risk is still not known.

The current study aimed at defining the protein stability and secondary structure distribution of the apoA-I protein in the lipid-bound HDL form, and to follow the dynamic protein structure changes in the lipid-binding process. Specifically, we aimed at determining if these parameters were affected in the protective Milano variant and in the atherogenic A164S variant and if that could explain their *in vivo* phenotypes. To achieve this, we used synchrotron radiation circular dichroism (SRCD) spectroscopy combined with native gel electrophoresis analyses of native/wild-type (WT), Milano and A164S apoA-I proteins in the lipid-free and lipid-bound states. SRCD was specifically chosen over conventional CD spectroscopy as the intense energy in SRCD spectroscopy allows for superior signal-to-noise ratio with negligible influence of the phospholipids on the signal in real-time, and thus for the determination of the secondary structure elements in both the presence and in the absence of phospholipids in a time-dependent manner^25^.

## Results

**Figure 1:**
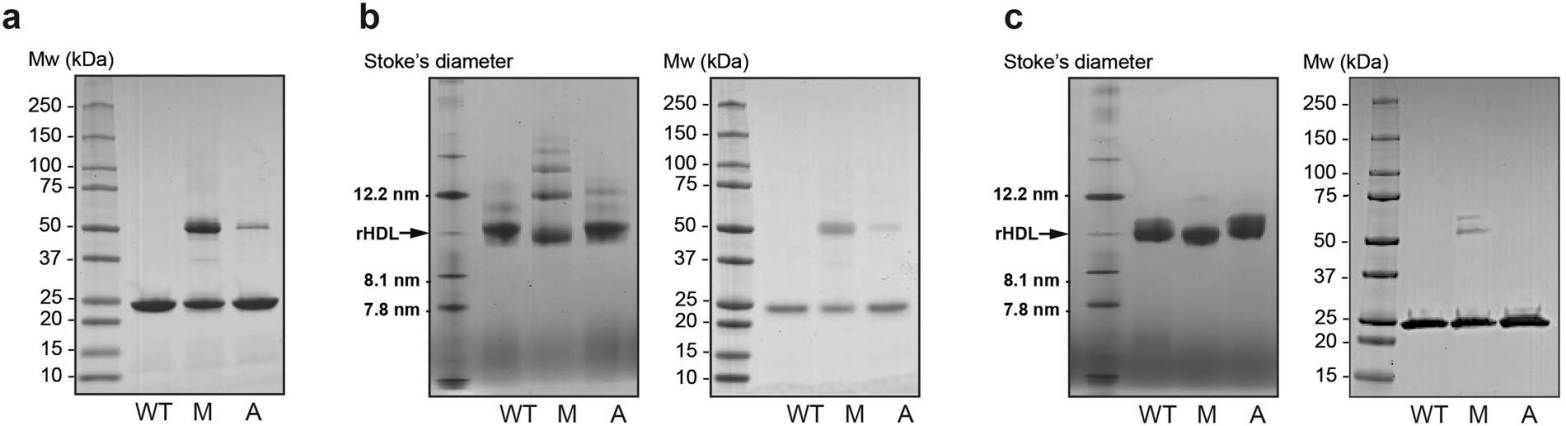
ApoA-I proteins analyzed in the lipid-free and lipid-bound states. Wildtype (*WT*), Milano (*M*) and A164S (*A*) apoA-I proteins were analyzed in the lipid-free (**a**) and lipid-bound (**b,c**) states. (**a**) SDS-PAGE analysis of proteins’ homogeneity before HDL formation. (**b,c**) Native gel analysis (*left panel*) and SDS-PAGE analysis (*right panel*) of proteins in the lipid-bound state before (**a**) and after (**b**) size exclusion chromatography. 2.5 µg of protein was loaded per lane in both SDS-PAGE and native gel analysis.

Human apoA-I proteins (WT, Milano, and A164S) were produced in a bacterial system, purified by affinity chromatography and treated with TEV protease to remove the affinity purification tag. The purified proteins (Fig. 1a) were incubated with DMPC phospholipids to form HDL particles (Fig. 1b). HDL particles of comparable sizes were produced although the Milano variant formed an array of HDL sizes and a higher degree of oligomerization; this was seen also at reducing and denaturing conditions (Fig. 1a and right panel in Fig. 1b). Size exclusion chromatography (SEC) was then performed in order to isolate 9.6 nm HDL particles (Fig. 1c and Fig. S1), which resulted in highly homogenous HDL populations (Fig. 1c, left panel) composed of proteins that were monomeric at reducing and denaturing conditions (Fig. 1c, right panel).

**Figure 2:**
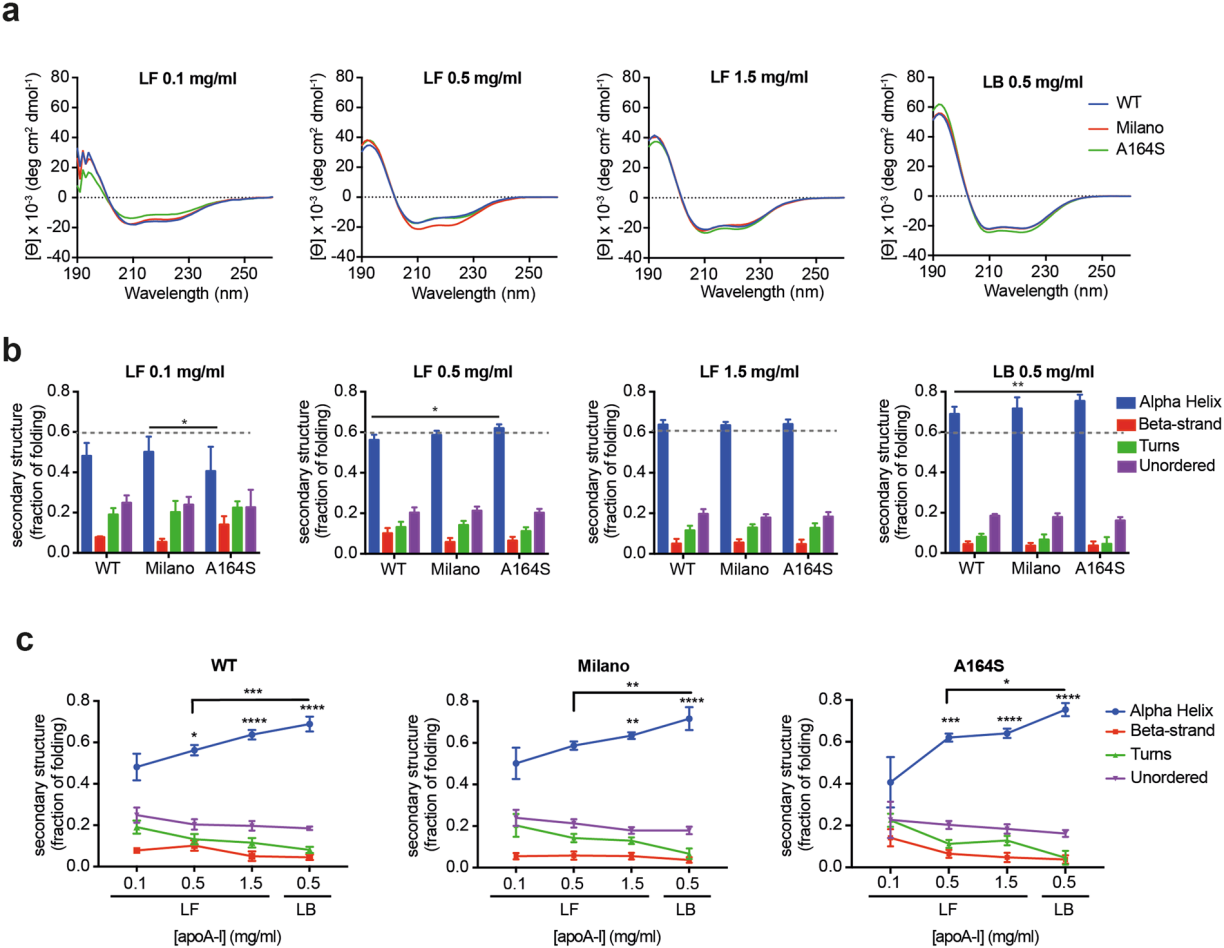
SRCD spectroscopy determines concentration and lipid-state dependent secondary structure distribution in apoA-I WT, Milano and A164S proteins. (**a**) Soluble proteins (McIlvaine Buffer) were scanned by SRCD spectroscopy (in the range of 190–260 nm) at indicated protein concentrations (0.1 mg/ml, 0.5 mg/ml and 1.5 mg/ml for lipid-free (*LF*) proteins, and 0.5 mg/ml for lipid-bound (*LB*) protein in 9.6 nm HDL particles). (**b**,**c**) The obtained spectra were used for determination of the relative secondary structure distribution (alpha helix, beta strand, turns and unordered) of the proteins. Graphs are shown to either highlight the inter-variant similarities/differences at a given protein concentration and lipidation-state (**b**), or to visualize the intra-variant protein concentration and lipid-state dependent secondary structure distribution (**c**). Data is shown as mean ± SD; significance is calculated according to two-way ANOVA (* p < 0.05, **p < 0.005, ***p < 0.001, ****p < 0.0001).

SRCD was used to compare the secondary structure distribution between WT, Milano and A164S apoA-I proteins at different protein concentrations (0.1 mg/ml, 0.5 mg/ml and 1.5 mg/ml) to reflect monomeric (0.1 mg/ml), dimeric/tetrameric (0.5 mg/ml), and tetrameric/higher order of oligomerization (1.5 mg/ml)^18,26–28^, and in the lipid-bound HDL state (Fig. 2). Importantly, as the lipids are essentially “invisible” in the SRCD analyses (Fig. S2) (the minor contribution of lipids, less than 5% of the protein signal, were subtracted) the observed changes in amplitude relates specifically to the lipid-bound protein structure.

Spectra were obtained in the region 190–260 nm (Fig. 2a) followed by deconvolution and estimation of the relative content of alpha-helix, beta strand, turn and unordered secondary structures, respectively (Fig. 2b). The alpha-helical content of the apoA-I WT protein increased in a concentration-dependent manner as previously described^27^ with a secondary structure distribution of 48% alpha-helix, 8% beta strand, 19% turns and 25% unordered structure in the monomeric state (0.1 mg/ml), and 63% alpha-helix, 5% beta strand, 12% turns and 20% unordered structure at a protein concentration of 1.5 mg/ml (multimeric assemblies). In the lipid-bound state the alpha-helix content was 69% (beta strand 4.5%, turn 8% and unordered 18%), which is lower than that previously observed by conventional CD spectroscopy (~76–78% alpha helical^29^). The secondary structure distributions of the Milano and A164S proteins were similar to the WT protein, with two main exceptions in the case of the A164S protein. The A164S variant had a lower helical content at 0.1 mg/ml than WT and Milano, and higher alpha helical content in the lipid bound form (alpha helix is 75% in A164S vs 69% and 71% in WT and Milano, respectively). No significant differences in the secondary structure composition of WT and Milano were observed when comparing the heterogeneous lipid-bound samples before SEC with the 9.6 nm particles obtained after SEC (Fig. S3).

**Figure 3:**
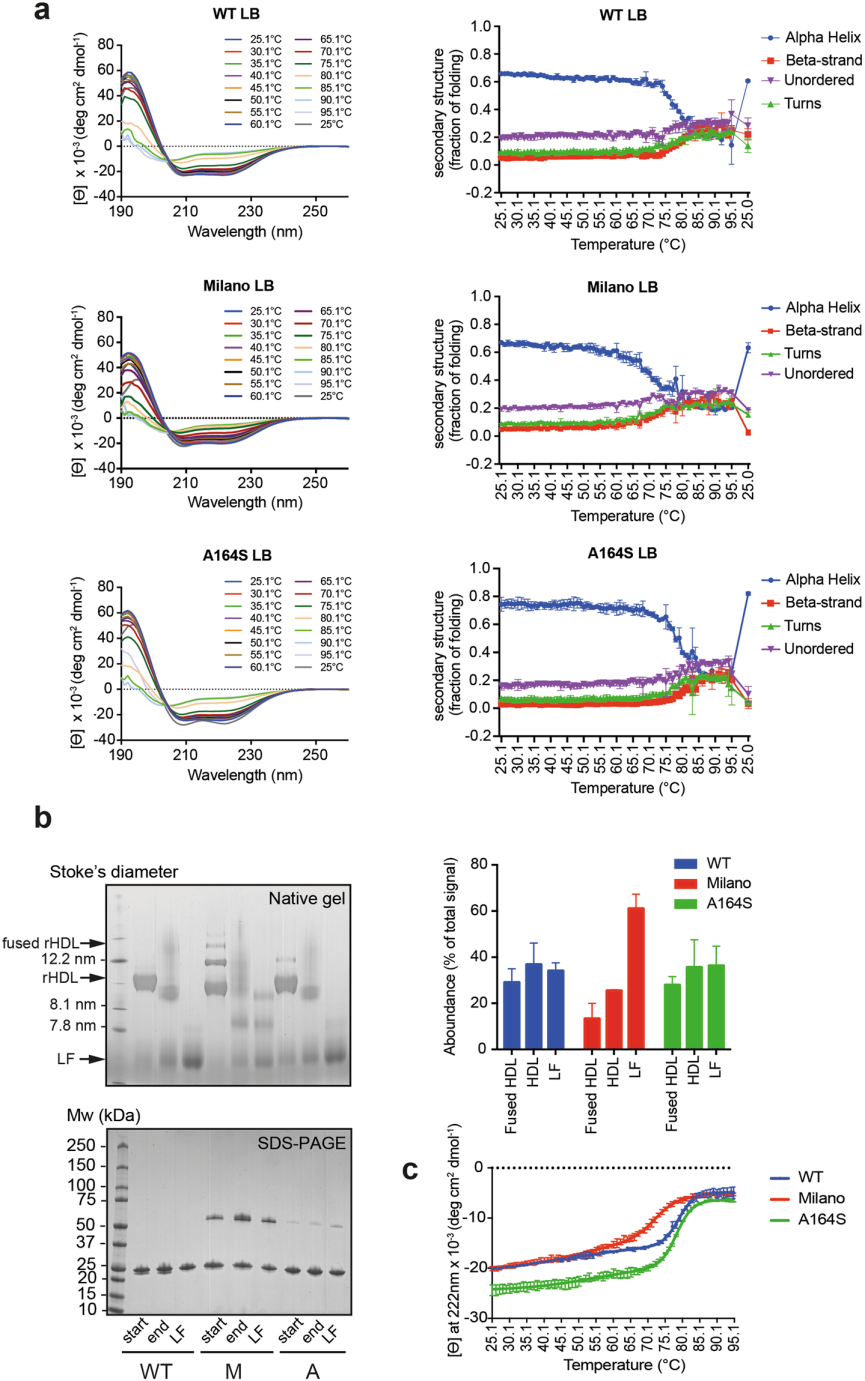
Thermal denaturation of apoA-I WT, Milano and A164S proteins in HDL. (**a**) Lipid-bound (*LB*) WT, Milano and A164S proteins in HDL were scanned by SRCD spectroscopy (in the range of 190–260 nm) in the temperature range 25.1 °C to 95.1 °C, with increments of 1 °C followed by cooling to 25 °C (*left panel*). Spectra were analyzed to determine the temperature-dependent relative secondary structure distribution (alpha helix, beta strand, turns and unordered) of the proteins (*right panel*). (**b**) Native gel analysis was used to analyze the size distribution of the WT (*W*), Milano (*M*) and A164S (*A*) HDL particles before (*start*) and after (*end*) the thermal cycle procedure. Lipid-free proteins were included as controls (*LF*) in the analysis. 8 µg (Native PAGE) or 2.5 µg (SDS-PAGE) of protein was loaded per lane. The species obtained upon thermal denaturation were quantified and the amount of each species expressed as percentage with respect to the total signal (*right pane*l). (**c**) The SRCD signal amplitude at 222 nm, which reflects the alpha helix content, was used to monitor the thermal unfolding process of the lipid-bound proteins (WT, Milano, A164S) as a function temperature. Data is shown as mean ± SD.

The Milano variant has previously been shown to have a reduced thermal stability in the lipid-free state^30^, whereas the stability of the lipid-free A164S variant was shown to be comparable to the lipid-free WT protein^31^. To determine the protein stability of the three proteins in the lipid-bound state, thermal denaturation combined with SRCD was used. The SRCD spectra were obtained in the temperature range 25 °C to 95 °C and then cooled down to 25 °C, followed by analyses of the changes in secondary structures (Fig. 3a). The alpha helical content decreased for all three proteins with higher temperature (to about 20% alpha helix at 95 °C), whereas in particular the beta strand and turns, and to a lesser extent the unordered structure, increased concomitantly. Interestingly, the denatured/renatured proteins retained their capabilities to bind lipids and to form HDL particles (Fig. 3b). Accordingly, the structural changes were reversible, with the alpha helical content being essentially restored to that before the heating cycle was initiated (Fig. 3a). It is also worth to notice that the amount of Milano variant that dissociated upon thermal unfolding was higher compared to that of the other apoA-I proteins (Fig. 3b, right panel). This observation might suggest a higher protein flexibility which is reflected in a reduced stability to thermal perturbation.

The amplitude at 222 nm was used as an indirect and specific measurement of the transition in the protein structure (Fig. 3c). The thermal denaturation followed a biphasic unfolding process that likely reflects the combination of structural reorganization of the apoA-I protein per se as well as protein-protein dissociation. The melting temperatures of the proteins indicated major inter-variant differences in the stability of the proteins (Table 1). In particular, the second phase unfolding of the Milano variant occurred at a lower temperature than for the WT and for the A164S variant. This is in line with the thermal denaturation of lipid-free Milano, which has a lower Tm than both WT and A164S (Table 1; all three lipid-free proteins exhibit monophasic melting) and suggests that the Milano protein structure is less stable in both the presence and absence of lipids. On the contrary, the A164S protein stability is essentially identical to the WT protein in their lipid-free states (Table 1)^31^, but, instead, show a much larger dynamic range in the protein stability in the lipid-bound state.

**Table 1:** Melting temperatures (Tm) calculated for the thermal unfolding of the ApoA-I proteins. Tm were calculated by fitting the experimental data with a biphasic non-linear regression. Data shown are the mean ± SD.

	lipid-bound (current study)*		Lipid-free
	Tm1	Tm2	Tm
ApoA-I WT	46.6 °C ± 3.6 °C	78.8 °C ± 0.2 °C	57.3 °C ± 0.4 °C^31^ 55.9 °C ± 1.4 °C^30^ 55.3 °C ± 0.5 °C^42^
ApoA-I Milano	55.9 °C ± 5.0 °C	72.15 °C ± 0.4 °C	50.9 °C ± 1.4 °C^30^
ApoA-I A164S	55.69 °C ± 6.1 °C	78.3 °C ± 0.2 °C	57.2 °C ± 0.2 °C^31^

*R square for the biphasic fitting of the experimental data points: 0.9914, 0.9916 and 0.9911 for WT, Milano and A164S apoA-I proteins, respectively.

In addition to studies on the apoA-I variants in their static lipid-free and lipid-bound states we also wished to follow the dynamics in the lipid-binding processes from a kinetic and structural point of view.

**Figure 4:**
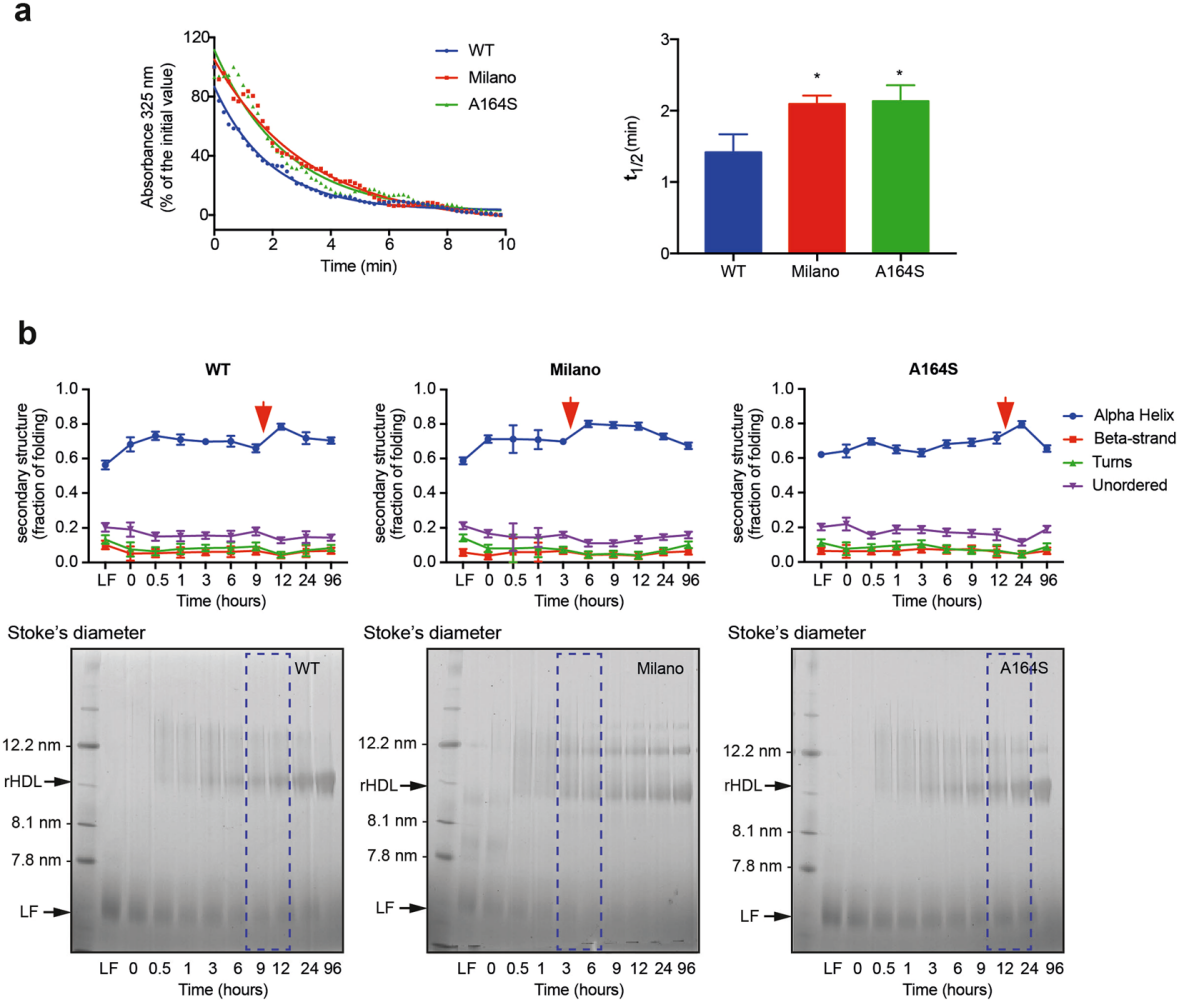
Structural transition of apoA-I WT, Milano and A164S proteins in lipid-binding and HDL formation. (**a**) Lipid Clearance Assay. Lipid-free apoA-I WT, Milano and A164S proteins were incubated with multilamellar DMPC vesicles with a 1:100 lipid to protein molar ratio and the decrease of the absorbance at 325 nm was measured every 10 s for 10 min. Experimental points were fitted to one-way decay of non-linear regression and the rates of lipid binding (t_1/2_) were calculated. Data shown are the mean ± SEM of 3 independent experiments carried out in triplicate. Significance is calculated according to one-way ANOVA (*p < 0.05). (**b**) Lipid-free apoA-I WT, Milano and A164S proteins were mixed with DMPC phospholipids (1:100 molar ratio) and scanned by SRCD spectroscopy (in the range of 190–260 nm) at the indicated times of incubation. Secondary structure distribution (alpha helix, beta strand, turns and unordered) of the proteins was estimated by deconvolution of the obtained spectra and plotted as a function of time (*top panel*). Native gel analysis was used to analyze formation and size distribution of the WT, Milano and A164S HDL particles during the time-dependent lipidation process (*bottom panel*). Lipid-free (*LF*) proteins were included for comparison. *Red arrows* indicate delayed increases in alpha helix secondary structure content. 1.5 µg of protein was loaded per lane. Data is shown as mean ± SD.

First, a lipid clearance assay was used to assess the lipid binding capacity of the apoA-I proteins (Fig. 4a). The rate of DMPC vesicles clearance was expressed as t_1/2_, calculated by fitting the experimental data with a one-way decay curve of non-linear regression. Interestingly, both Milano and A164S variants showed a slower lipid binding kinetics with a 1.5-fold increase in the t_1/2_, compared to WT (Fig. 4a, right panel).

Then, SRCD was used to determine the structure dynamics in the lipidation process. As described above, this technique is highly suitable for this type of analyses as the lipids do not contribute significantly to the obtained signal. Following mixing of proteins and lipids, spectra were acquired for up to 96 hours. In addition to the 0 h time point (effectively this is the 3–5 min time point due to time required for mixing, loading and for scanning) we also included the lipid-free data in the comparison. Interestingly, the initial structural changes occurred essentially instantly for the WT and Milano proteins (compare the LF and 0 h time point values in Fig. 4a), whereas substantial changes in the A164S structure occurred later in the HDL formation process. The instant changes (LF to 0 h) in secondary structure were not accompanied by any apparent formation of HDL particles (see gels in Fig. 4b) suggesting that partial lipidation of apoA-I and the concurrent transition to alpha helical structure results in less defined intermediate conformational states prior to the final formation of HDL particles. We could also note that the initial increase in alpha helix structure (LF to 0 h) was first followed by a plateau and then a second increase in alpha helix content (arrows in Fig. 4). This second increase emerged at different lengths of incubation time for the different proteins (Milano < WT < A164S). The significance of this observation is not clear but may reflect a higher capability of the Milano structure to adapt to the lipid-cargo, and conversely a reduced capability of the A164S, compared to the WT protein. Native gel analysis of the protein-lipid complexes at the different time-points were performed to assess if also qualitative differences could be observed. The time-dependent formation of HDL showed clear variant-specific differences where the Milano protein, and to a lower degree the A164S protein, formed HDL particle subpopulations of larger sizes (about 12 nm in diameter) in addition to the major 9.6 nm HDL population. However, the second increase in the alpha helical content of the proteins was not accompanied by an apparent change in HDL size distribution (compare 9 h and 12 h for WT, 3 h and 6 h for Milano, and 12 h and 24 h for A164S, respectively, in the native gels in Fig. 4) suggesting that the secondary structure conversion occurred in HDL particle without changes in the particle dimensions. Similarly, the conversions seemed not to reflect the variant-specific lipid-binding in the lipid-clearance assay (Fig. 4) suggesting that the two events are not directly connected.

## Discussion

As already discussed in the Introduction section, apoA-I has a central role in the many functions of HDL in lipid and glucose transport and metabolism. Therapeutic approaches based on the apoA-I biology in cardiovascular and metabolic diseases are therefore explored. The apoA-I protein is also an attractive model to study protein structure dynamics in the formation of lipid-protein complexes, which is facilitated by abundant protein access either from blood plasma or from heterologous production of apoA-I, as well as the relatively small size of the protein (243 amino acids in the mature apoA-I) and the large conformational switch that occurs in the lipid-binding process. Yet, there are limitations in our knowledge about the dynamic behavior of the protein structure, and we also do not have complete knowledge on how clinically relevant amino acid substitutions and modifications affect protein structure and function.

To shed light on this, we have here explored the static and dynamic structures of variants of apoA-I that are known to be protective (R173C; Milano) or atherogenic (A164S) by comparative studies of the proteins in the apo-state, in the lipid-bound HDL-state as well as during the lipid-binding and formation of nascent HDL. SRCD spectroscopy was selected as the preferred method as it allows for in-solution studies on protein secondary structure distribution and also to monitor real-time conformational changes. This method is particularly useful for proteins with high structure plasticity, including intrinsically disordered proteins (e.g., see ref.^32^) and lipid-binding lipoproteins (e.g., see ref.^33^). A great advantage with SRCD is that the intense light provided by the synchrotron radiation highly reduces the background absorbance from the buffer constituents and also from the phospholipids, which otherwise is a major obstacle in conventional bench-top CD analyses (Fig. S2)^25^. This is of particular importance for the 180 nm to 200 nm wavelength region, which is needed for reliable estimation of the relative secondary structure composition of the protein (spectra in the range 180–260 nm is used), and absolutely crucial when studying conformational changes during HDL formation in the presence of phospholipids.

As was expected all three variants formed DMPC-rHDL particles with a diameter of about 9.6 nm. In addition, a range of larger particle sizes was also formed with the Milano protein (as shown by native gel analyses, Fig. 1b), which likely is due to a disulfide bond between cysteines. The alpha helical content of the WT rHDL particles (69%) was found to be less than expected when comparing with the X-ray crystal structures of truncated apoA-I proteins^10,11^; both truncated proteins were crystallized in the absence of lipids but based on their open, circular confirmations they have been assumed to be in a lipid-bound resembling state^34^, which present a larger degree of helical content. However, as is shown here the secondary structure content of the lipid-free apoA-I increases with protein concentration (Fig. 2b) and also by a crowded environment^27^. It is therefore plausible that the high protein concentration in the protein crystals may induce helix-formation in the apoA-I structure that does not fully reflect the dynamic structure in solution. Here, the SRCD analyses of the HDL particles are performed in solution and at a protein concentration of 0.5 mg/ml. A helical content of about 69–75% in the 9.6 nm rHDL particles corresponds to about 175 amino acid residues, which is sufficient to enwrap the phospholipid bilayer in the 9.6 nm discs. The SRCD data also define a substantial amount of beta strand structure (3.8–4.5% beta strand structure), corresponding to about 11 amino acids. This is comparable to previous findings based on EPR spectroscopy analyses defining region 40–50, and 149–157, as beta strand structure in the 9.6 nm HDL particles^20^. The stretch of beta strand structure in region 149–157, which was also defined as a flexible, two-state region in a hydrogen-deuterium exchange mass-spectroscopy study^17^, would provide additional length to the circumference (alpha helix extends 1.5 Å per amino acid, and beta strand structure extends 3.5 Å per amino acid) and potentially allow for disc size expansion.

The thermal stability of the three proteins in the lipid-bound HDL state varied. Proteins followed a biphasic melting curve, with similar stabilities at the lower temperatures, but with reduced thermal stability for the Milano variant at higher temperatures. The relation with a reduced stability of the Milano protein in the HDL state compared to WT is similar to that previously shown in comparative studies of the thermal stability of the lipid-free counterparts (Table 1)^30^. The reason for this is not clear but the finding may suggest that a destabilization of the apoA-I structure in both the lipid-free and lipid-bound state, with increased dynamic behavior, facilitates the function of the protein.

Similarly, an increased destabilization would potentially also affect the exchange between lipid-associated and lipid-free state as was previously shown by Cavigiolio and colleagues by comparing oxidized and native apoA-I protein^35^. In support of this, our studies show that the lipid-free proteins rapidly, within minutes, adopt a conformation that has high alpha-helical secondary structure content, before any visible formation of HDL particles form is seen. This is particularly pronounced for the WT and Milano proteins, but to a lesser extent in the A164S protein. Interestingly, the apparent reformation of the apoA-I structure in the HDL particles (arrows in Fig. 4) occur much earlier in time for the Milano variant.

The introduction of a hydrophilic serine instead of the alanine in the A164S protein was previously shown to have no effect on protein stability in the lipid-free state^31^. Taking this into account, and that HDL formation generally stabilizes the apoA-I structure, it was therefore a bit surprising that this modification decreased the stability of the A164S protein in the HDL particle. However, in the Mei and Atkinson X-ray crystal structure^11^ the Ala164 side-chain points towards the interior of the phospholipid bilayer, and the introduction of a hydrophilic amino acid at this position would thus be unfavorable for the stability of the complex. An impaired interaction of the A164S apoA-I variant with phospholipids is also suggested by the reduced efficiency of the A164S protein to bind lipids in a lipid clearance assay (Fig. 4a)^31^.

In conclusion, our data propose that the R173C (Milano) and A164S amino acid substitutions both lead to reduced protein stability in the HDL bound state. However, while the destabilization of the Milano variant is favorable for protein function, the decreased stability of the A164S variant in the HDL particles appears to negatively affect protein functionality. These findings stress the importance of a fine balance of protein structure stability in lipid-binding and exchange, and is informative for the evaluation of compounds aimed at increasing HDL efficacy.

## Methods

### Protein production and purification

ApoA-I WT, Milano (R173C) and A164S proteins were produced and purified as previously described^30,31^. In short, a bacterial expression system consisting of pEXP-5 plasmid (Novagen) in *Escherichia coli* strain BL21(DE3) pLysS cells (Invitrogen) was used to produce the apoA-I proteins. His-tagged ApoA-I proteins were purified from bacterial cell lysate by immobilized metal affinity chromatography (His-Trap-Nickel-chelating columns, GE Healthcare) under denaturing conditions (3 M guanidine in phosphate-buffered saline (PBS), pH 7.4). Following binding, an extensive wash with 40 mM imidazole in PBS was performed and proteins were then eluted with 500 mM imidazole in PBS. Imidazole was removed from protein samples by using desalting columns (GE Healthcare) equilibrated with PBS, pH 7.4, and tobacco etch virus (TEV) protease was employed overnight at 4 °C, in the presence of 1 mM DTT, to remove the His-tag from protein samples. At the end of the incubation, a reverse Ni^2+^-column step was employed in order purify the cleaved ApoA-I from TEV protease and the His-tag. The flow-through containing cleaved ApoA-I protein was desalted into McIlvaine Buffer (165 mM Na_2_HPO_4_, 17.6 mM citrate, pH 7) and stored at 4 °C prior to use. SDS-PAGE and Blue-native gel (Invitrogen) were used for analyses of lipid-free protein and HDL particles.

### Lipid clearance assay

Lipid clearance was monitored by monitoring the decrease in the absorbance of the multilamellar vesicles and protein mixture at 325 nm every 10 s for 10 minutes, as previously described^31^.

### HDL formation

1,2-dimyristoyl-*sn*-glycero-3-phosphocholine (DMPC) (Avanti Polar Lipids, Alabaster, AL, USA) was dissolved in chloroform:methanol (3:1) which was evaporated under a stream of nitrogen gas and the resulting lipid film was resuspended in McIlvaine buffer. The DMPC emulsion was passed through a polycarbonate membrane with 100 nm pore size using the LiposoFast system (Avestin, Ottawa, ON, Canada). The resulting vesicles were incubated with apoA-I at a 100:1 molar ratio (phospholipid:protein) at 24 °C, in agitation. Samples were taken at 0, 0.5, 1, 3, 6, 9, 12, 24, 96 h and stored at −80 °C in the presence of 10% sucrose prior the Blue Native PAGE analysis, or analysed by SRCD.

To isolate the 9.6 nm DMPC lipoparticles, a size-exclusion chromatography (SEC) was employed. SEC was performed by using a preparative Superose 6 increase 10/300 GL column (GE Healthcare) and samples were eluted in McIlvaine buffer, pH 7, at a flow of 0.5 ml/min. Isolates species were analysed by Native Page, followed by Coomassie staining and Western blot analysis, by using ApoA-I antibodies (Q0496, DAKO).

### SRCD measurements

SRCD experiments were performed using a nitrogen-flushed Module B end-station spectrophotometer, equipped with a 6-cell turret, at B23 Synchotron Radiation CD Beamline at the Diamond Light Source^36–38^. For the concentration dependence studies, lipid free (LF) ApoA-I protein samples were dialysed against McIlvaine buffer in order to minimize the noise at wavelengths below 200 nm. Protein samples were diluted at 0.1, 0.5 and 1.5 mg/ml, loaded into quartz cuvettes (0.1, 0.2 or 0.5 mm path lengths, depending on protein concentration) and the spectra acquired at 25 °C in the far-UV range 185–260 nm, with a 1 nm wavelength increment. Lipid-bound (LB) samples were produced in McIlvaine buffer and analysed by SRCD at 0.5 mg/ml in a 0.2 nm quartz cuvette. All the spectra were corrected subtracting the background signal of the buffer (McIlvaine Buffer, or DMPC vesicles in McIlvaine Buffer, depending on sample). Secondary structure estimation from CD spectra was carried out using CDApps^39^ using Continll algorithm with reference data SP 43^40^. The molar ellipticity ([Θ]) was calculated according to the equation described in^41^.

### Conventional CD measurements

Concentional CD measurements were performed on a Jasco J-810 spectropolarimeter equipped with a Jasco CDF-426S Peltier, set to 25 °C. Samples were loaded into a 1 mm quartz cuvette and the spectra acquired at 25 °C in the far-UV range 190–260 nm, with a 1 nm wavelength increment.

### Thermal stability analyses of LB proteins

LB proteins were diluted in McIlvaine buffer at 0.5 mg/ml and placed in a 0.2 mm quartz cuvette. SRCD spectra were collected in 25.1–95.1 °C range, with a 1 °C increment. Results were processed using CDApps^39^ and Tm estimated by biphasic fitting using GraphPad Prism 7. The analysis of the secondary structure during the thermal denaturation was performed by using the CONTINLL algorithm with reference data SP 43, between 190 and 260 nm.

A PCR thermal cycler (TC-Plus, Techne, Staffordshire, UK) was used to denature/renature samples for electrophoretic analysis. The quantification of the species visualized on the gel was performed by using ImageJ software and the amount of each species was plotted as fold change with respect to the total signal.

## Electronic supplementary material

Supplementary data
