# Supplementary material for: Synchrotron radiation circular dichroism spectroscopy reveals structural divergences in HDL-bound apoA-I variants
Source: Sci Rep. 2017 Oct 19;7:13540. doi: 10.1038/s41598-017-13878-z (PMC5648894; doi:10.1038/s41598-017-13878-z)
Supplement: Supplementary file 1 — Supplementary data [file 41598_2017_13878_MOESM1_ESM.doc]

**Supplementary Figures to:**

**Synchrotron radiation circular dichroism spectroscopy reveals structural divergences in HDL-bound apoA-I variants.**

Rita Del Giudice, Oktawia Nilsson, Joan Domingo-Espín and Jens O. Lagerstedt*

Department of Experimental Medical Science, Lund University, 221 84 Lund, Sweden

*Corresponding Author. Email: jens.lagerstedt@med.lu.se ; Phone: +46-46-2227266

**Supplementary Figure S1**

**Fig S1. Isolation of 9.6 nm DMPC lipoparticles.** DMPC-protein mixtures were incubated at 24°C for 96 hours and the 9.6 nm DMPC particles isolated by SEC. (**a**) Elution profiles of WT, Milano and A164S protein-DMPC mixture. (**b**) Native PAGE of the isolated species analyzed by Coomassie staining (*left panel*) and western blot (*right panel*). 2 µg of protein was loaded per lane.

**Supplementary Figure S2**

**Fig S2. Negligible SRCD signal from citrate buffer and DMPC phospholipids.** Citrate buffer in the presence (*DMPC*) or absence (*citrate buffer*) of DMPC phospholipids were scanned by (**a**) SRCD or (**b**) conventional CD spectroscopy, in the range of 190-260 nm, and compared with the signal amplitude of lipid-free apoA-I WT protein at 0.5 mg/ml concentration (*WT LF apoA-I 0.5 mg/ml*).

**Supplementary Figure S3**

**Fig S3. Comparison between heterogeneous DMPC-lipoparticles and isolated 9.6 nm DMPC lipoparticles.** DMPC-protein mixtures were incubated at 24°C for 96 hours, the 9.6 nm DMPC particles isolated by SEC, and samples analyzed by SRCD. (**a**) Native PAGE of the heterogeneous DMPC particles and isolated species analyzed by Coomassie staining (*left panel*) and western blot (*right panel*). 2 µg of protein was loaded per lane. (**b**) Protein samples were scanned by SRCD spectroscopy (in the range of 190-260 nm) at 0.5 mg/ml and the obtained spectra used for determination of the relative secondary structure content (**c**). Data is shown as mean±SD; significance is calculated according to two-way ANOVA (** p<0.005, *** p<0.001).
